# Do the Expert Recommendations for Implementing Change (ERIC) strategies adequately address sustainment?

**DOI:** 10.3389/frhs.2022.905909

**Published:** 2022-11-03

**Authors:** Nicole Nathan, Byron J. Powell, Rachel C. Shelton, Celia V. Laur, Luke Wolfenden, Maji Hailemariam, Sze Lin Yoong, Rachel Sutherland, Melanie Kingsland, Thomas J. Waltz, Alix Hall

**Affiliations:** ^1^Hunter New England Population Health, Hunter New England Area Health Service, Newcastle, NSW, Australia; ^2^School of Medicine and Public Health, The University of Newcastle, Newcastle, NSW, Australia; ^3^Priority Research Centre for Health Behaviour, The University of Newcastle, Newcastle, NSW, Australia; ^4^Hunter Medical Research Institute, New Lambton Heights, Newcastle, NSW, Australia; ^5^Center for Mental Health Services Research, Brown School, Washington University in St. Louis, St. Louis, MO, United States; ^6^Division of Infectious Diseases, John T. Milliken Department of Medicine, Washington University School of Medicine, Washington University in St. Louis, St. Louis, MO, United States; ^7^Center for Dissemination and Implementation, Institute for Public Health, Washington University in St. Louis, St. Louis, MO, United States; ^8^Department of Sociomedical Sciences, Columbia University, Mailman School of Public Health, New York, NY, United States; ^9^Women's College Hospital Institute for Health System Solutions and Virtual Care, Toronto, ON, Canada; ^10^Institute of Health Policy, Management and Evaluation, University of Toronto, Toronto, ON, Canada; ^11^Department of Obstetrics, Gynaecology and Reproductive Biology, College of Human Medicine, Michigan State University, East Lansing, MI, United States; ^12^School of Health Science, Swinburne University of Technology, Hawthorn, VIC, Australia; ^13^Department of Psychology, Eastern Michigan University, Ypsilanti, MI, United States

**Keywords:** sustainability, sustainment, implementation strategies, mechanisms, design and tailoring, implementation science

## Abstract

**Background:**

Sustainability science is an emerging area within implementation science. There is limited evidence regarding strategies to best support the continued delivery and sustained impact of evidence-based interventions (EBIs). To build such evidence, clear definitions, and ways to operationalize strategies specific and/or relevant to sustainment are required. Taxonomies and compilations such as the Expert Recommendations for Implementing Change (ERIC) were developed to describe and organize implementation strategies. This study aimed to adapt, refine, and extend the ERIC compilation to incorporate an explicit focus on sustainment. We also sought to classify the specific phase(s) of implementation when the ERIC strategies could be considered and applied.

**Methods:**

We used a two-phase iterative approach to adapt the ERIC. This involved: (1) adapting through consensus (ERIC strategies were mapped against barriers to sustainment as identified *via* the literature to identify if existing implementation strategies were sufficient to address sustainment, needed wording changes, or if new strategies were required) and*;* (2) preliminary application of this sustainment-explicit ERIC glossary (strategies described in published sustainment interventions were coded against the glossary to identify if any further amendments were needed). All team members independently reviewed changes and provided feedback for subsequent iterations until consensus was reached. Following this, and utilizing the same consensus process, the Exploration, Preparation, Implementation and Sustainment (EPIS) Framework was applied to identify when each strategy may be best employed across phases.

**Results:**

Surface level changes were made to the definitions of 41 of the 73 ERIC strategies to explicitly address sustainment. Four additional strategies received deeper changes in their definitions. One new strategy was identified: *Communicate with stakeholders the continued impact of the evidence-based practice*. Application of the EPIS identified that at least three-quarters of strategies should be considered during preparation and implementation phases as they are likely to impact sustainment.

**Conclusion:**

A sustainment-explicit ERIC glossary is provided to help researchers and practitioners develop, test, or apply strategies to improve the sustainment of EBIs in real-world settings. Whilst most ERIC strategies only needed minor changes, their impact on sustainment needs to be tested empirically which may require significant refinement or additions in the future.

## Introduction

Over the last two decades, research investment in, and application of, implementation science theories, frameworks and methods has resulted in significant improvements in the initial implementation of evidence-based interventions (EBIs) in both clinical and community settings (1–3). Key to advancing the field has been the concerted efforts, particularly in the last few years, to identify effective implementation strategies (and the mechanisms through which they operate) (4–7). Implementation strategies are “*methods or techniques used to improve the adoption, implementation, sustainment and scale-up of interventions.”* (3, 8), Systematic reviews of implementation trials have assessed the impact implementation strategies have had on the adoption and implementation of EBIs in real world settings (2, 3, 9–11).

Poor and inconsistent reporting of implementation strategies has been a longstanding issue for the field (8). Historically, the language used to define implementation strategies has been inconsistent and highly variable (12, 13), with different terms used to describe the same strategy or the same terms being used to define different strategies (13, 14). Consequently, descriptions of implementation strategies have lacked the necessary detail required for an adequate understanding of the exact nature, function, and make-up of an implementation intervention (i.e., combination of one or more implementation strategies used to support the delivery of an evidence-based practice, program or intervention) (12, 14–16). Such information is essential for scientific advancement, as it allows for replication in advancing the science and improvements of previous research, as well as for scale-up and translation of effective strategies into practice beyond the initial site (14). These inconsistencies make it difficult to identify core functions of the implementation intervention or the implementation strategies, to synthesize research findings, and ultimately identify the active components of a particular implementation intervention. This problem is especially true for complex, multicomponent implementation interventions such as those typically employed in clinical and public health (14).

The introduction and application of taxonomies or compilations of implementation strategies and behavior change techniques is one approach that has been used to address such issues (12, 13, 17–20). Compilations standardize the naming and definitions of implementation strategies, enabling implementation interventions to be described in a consistent manner. A number of implementation-specific taxonomies and compilations have been developed to standardize and clarify the classification and reporting of implementation strategies (8, 11, 13, 17–19). The *Expert Recommendations for Implementing Change* (ERIC) compilation (8, 13) has been widely used in health and public health and has provided much-needed common terminology for implementation strategies. Developed and refined by implementation experts, the compilation shows high face validity and consists of 73 strategies grouped into nine categories (see Table 1) (21).

**Table 1:** Sustainment-explicit expert recommendations for implementing change (ERIC) (8–13) glossary.

**Conceptual strategy category from original ERIC compilation (20)**	**Strategy number from original ERIC compilation (20)**	**Strategy name**	**Strategy definition (8, 13)**	**Specific phase(s) when the strategies could be considered and applied**
Use evaluative and iterative strategies	4	Assess for readiness and identify barriers and facilitators	Assess various aspects of an organization and the broader context to determine its degree of readiness to implement and sustain, barriers that may impede implementation and sustainment, and strengths that can be used in the implementation and sustainment effort	Preparation, implementation and Sustainment
	5	Audit and provide feedback	Collect and summarize clinical performance data over a specified time period and give it to clinicians and administrators to monitor, evaluate, and modify provider behavior	Preparation, Implementation and Sustainment
	New sustainment strategy	Communicate with stakeholders the continued impact of the EBP	Communicate data to external stakeholders, end-users and consumers to demonstrate the ongoing benefit, cost effectiveness or return on investment of the innovation with continued implementation.	Implementation, Sustainment
	14	Conduct cyclical small tests of change	Implement changes in a cyclical fashion using small tests of change before taking changes system-wide. Tests of change benefit from systematic measurement, and results of the tests of change are studied for insights on how to do better. This process continues serially over time, and refinement is added with each cycle	Implementation and Sustainment
	18	Conduct local needs assessment	Collect and analyze data related to the initial and ongoing need for and fit of the innovation	All phases
	23	Develop a formal implementation blueprint	Develop a formal implementation blueprint that includes all goals and strategies. The blueprint should include the following: (1) aim/purpose of the implementation; (2) scope of the change (e.g., what organizational units are affected); (3) timeframe and milestones; and (4) appropriate performance/progress measures; (5)plan for maintenance and sustainment of the EBI once it has been implemented. Use and update this plan to guide the implementation effort over time	Preparation, Implementation and Sustainment
	61	Stage implementation scale up	Phase implementation efforts by starting with small pilots or demonstration projects and gradually move to a system wide rollout while sustaining delivery of the EBP in the original sites	Implementation, Sustainment
	26	Develop and implement tools for quality monitoring	Develop, test, and introduce into quality-monitoring systems the right input—the appropriate language, protocols, algorithms, standards, and measures (of processes, patient/consumer outcomes, and implementation outcomes) that are often specific to the innovation being implemented and sustained	Preparation, Implementation and Sustainment
	27	Develop and organize quality monitoring systems	Develop and organize systems and procedures that monitor clinical processes and/or outcomes for the purpose of quality assurance and improvement	Preparation, implementation and sustainment
	46	Obtain and use patients/consumers and family feedback	Develop strategies to increase patient/consumer and family feedback on the implementation and sustainment effort	Preparation, implementation and Sustainment
	56	Purposely reexamine the implementation	Monitor progress and adjust clinical practices and implementation strategies to continuously improve the quality of care	Implementation and Sustainment
	8	Centralize technical assistance	Develop and use a centralized system to deliver technical assistance focused on implementation and sustainment issues	Preparation and Implementation and Sustainment
Provide interactive assistance	33	Facilitation	A process of interactive problem solving and support that occurs in a context of a recognized need for improvement and a supportive interpersonal relationship	All phases
	53	Provide clinical supervision	Provide clinicians with ongoing supervision focusing on the innovation. Provide training for clinical supervisors who will supervise clinicians who provide the innovation	Implementation and Sustainment
	54	Provide local technical assistance	Develop and use a system to deliver technical assistance focused on implementation and sustainment issues using local personnel	Preparation and Implementation and Sustainment
	51	Promote adaptability	Identify the ways a clinical innovation can be tailored to meet local needs and clarify which elements of the innovation must be maintained to preserve fidelity. Continue to assess and adapt the fit of the innovation to ensure that is appropriate and sustained if still relevant.	All phases
	63	Tailor strategies	Tailor the implementation or sustainment strategies to address barriers and leverage facilitators that were identified through ongoing data collection	Preparation, Implementation and Sustainment
	67	Use data experts	Involve, hire, and/or consult experts to inform management on the use of data generated by implementation and sustainment efforts	Preparation and Implementation and Sustainment
	68	Use data warehousing techniques	Integrate clinical records across facilities and organizations to facilitate implementation across systems, continually assess that they are still appropriate	Preparation, Implementation and Sustainment
	6	Build a coalition	Recruit, cultivate and maintain relationships with partners in the implementation and sustainment effort	All phases
Develop staekholder interrelationships	7	Capture and share local knowledge	Capture local knowledge from implementation sites on how implementers and clinicians made something work and continue to work in their setting and then share it with other sites	Implementation and Sustainment
	17	Conduct local consensus discussions	Include local providers and other stakeholders in discussions that address whether the chosen problem is important and whether the clinical innovation to address it is appropriate and continues to be appropriate	Exploration and Sustainment
	40	Involve executive boards	Involve existing governing structures (*e.g*., boards of directors, medical staff boards of governance) in the implementation and sustainment effort, including the review of data on implementation and sustainment processes	All phases
	47	Obtain formal commitments	Obtain written commitments from key partners that state what they will do to implement the innovation and how they will support sustainment if it has the intended beneficial effects	Preparation
	Extension of strategy #47 explicit to sustainment	Re-affirm formal commitments	Revisit the written commitments obtained from key partners that state what they will do to implement and sustain the innovation. Assess whether these commitments are being upheld and whether new commitments are required to help sustain the innovation	Sustainment
	52	Promote network weaving	Identify, build and maintain existing high-quality working relationships and networks within and outside the organization, organizational units, teams, etc. to promote information sharing, collaborative problem-solving, and a shared vision/goal related to implementing and sustaining the innovation	All phases
	64	Use advisory boards and workgroups	Create and engage a formal group of multiple kinds of stakeholders to provide input and advice on implementation and sustainment efforts and to elicit recommendations for improvements	All phases
	24	Develop academic partnerships	Partner with a university or academic unit for the purposes of shared and ongoing training and bringing relevant research skills to an implementation or sustainment project	All phases
	25	Develop an implementation glossary	Develop and distribute a list of terms describing the innovation, implementation, and stakeholders in the organizational change	Preparation and Implementation
	36	Identify early adopters	Identify early adopters at the local site to learn from their experiences with the practice innovation	Exploration, Preparation and Implementation
	Extension of strategy #36 explicit to sustainment	Identify successful sustainers	Identify successful sustainer at the local site to learn from their experiences with the practice innovation	Sustainment
	38	Inform local opinion leaders	Inform providers identified by colleagues as opinion leaders or “educationally influential” about the clinical innovation in the hopes that they will influence colleagues to adopt it	Preparation and Implementation
	Extension of strategy #38 explicit to sustainment	Re-engage with local opinion leaders	Periodically re-engage with providers identified by colleagues as opinion leaders or “educationally influential” about the importance of continuing to deliver the practice innovation in the hopes that they will influence colleagues to sustain its use	Sustainment
	35	Identify and prepare champions	Identify and prepare individuals who dedicate themselves to supporting, marketing, and driving through an implementation, overcoming indifference or resistance that the intervention may provoke in an organization and continue to support sustainment	Preparation and Implementation and Sustainment
	45	Model and simulate change	Model or simulate the change that will be implemented prior to implementation	Exploration and Preparation
	48	Organize clinician implementation team meetings	Develop and support teams of clinicians who are implementing the innovation and give them protected time to reflect on the implementation effort, share lessons learned, and support one another's learning	Preparation and Implementation and Sustainment
	57	Recruit, designate, and train for leadership	Recruit, designate, train and retrain as necessary, leaders for the change effort	Preparation and Implementation and Sustainment
	65	Use an implementation advisor	Seek guidance from experts in implementation and sustainability	All phases
	72	Visit other sites	Visit sites where a similar implementation or sustainment effort has been considered successful	Preparation, implementation and Sustainment
	15	Conduct educational meetings	Hold meetings targeted toward different stakeholder groups (e.g., providers, administrators, other organizational stakeholders, and community, patient/consumer, and family stakeholders) to teach them about the clinical innovation	Preparation and Implementation and Sustainment
Train and educate stakeholders	16	Conduct educational outreach visits	Have a trained person meet with providers in their practice settings to educate providers about the clinical innovation with the intent of changing the provider's practice	Implementation and Sustainment
	29	Develop educational materials	Develop and format manuals, toolkits, and other supporting materials in ways that make it easier for stakeholders to learn about the innovation and for clinicians to learn how to deliver the clinical innovation	Preparation
	Extension of strategy #29 explicit to sustainment	Review and update educational materials	Review manuals, toolkits, and other supporting materials on how to deliver the clinical innovation and ensure they continue to be appropriate. Update the resources based on changing scientific evidence as needed	Sustainment
	60	Shadow other experts	Provide ways for key individuals to directly observe experienced people engage with or use the targeted practice change/innovation	Implementation and Sustainment
	19	Conduct ongoing training	Plan for and conduct training in the clinical innovation in an ongoing way, including training of new staff and booster training for existing staff	Preparation and Implementation and Sustainment
	20	Create a learning collaborative	Facilitate the formation of groups of relevant stakeholders or organizations and foster a collaborative learning environment to improve implementation and sustainment of the clinical innovation	Preparation and Implementation and Sustainment
	31	Distribute educational materials	Distribute educational materials (including guidelines, manuals, and toolkits) in person, by mail, and/or electronically	Implementation and Sustainment
	43	Make training dynamic	Vary the information delivery methods to cater to different learning styles and work contexts, and shape the training in the innovation to be interactive	Preparation and Implementation and Sustainment
	55	Provide ongoing consultation	Provide ongoing consultation with one or more experts in the strategies used to support implementing and sustaining the innovation	Preparation and Implementation and Sustainment
	71	Use train-the-trainer strategies	Train designated personnel or organizations to train others in the clinical innovation	Implementation and Sustainment
	73	Work with educational institutions	Encourage educational institutions to train clinicians in the innovation	Preparation and Implementation and Sustainment
	21	Create new clinical teams	Change who serves on the clinical team, adding different disciplines and different skills to make it more likely that the clinical innovation is delivered (or is more successfully delivered) in an ongoing way	Preparation and Implementation and Sustainment
Support clinicians	30	Develop resource sharing agreements	Develop partnerships with organizations that have resources needed to implement and sustain the innovation	Preparation and Implementation and Sustainment
	32	Facilitate relay of clinical data to providers	Provide as close to real-time data as possible about key measures of process/outcomes using integrated modes/channels of communication in a way that promotes use of the targeted innovation	Implementation and Sustainment
	58	Remind clinicians	Develop, review and update reminder systems designed to help clinicians to recall information and/or prompt them to use the clinical innovation	Preparation and Implementation and Sustainment
	59	Revise professional roles	Shift and revise roles among professionals who provide care, and redesign job characteristics	Preparation and Implementation and Sustainment
	37	Increase demand	Attempt to influence the market for the clinical innovation to increase competition intensity and to increase the maturity of the market for the clinical innovation	Preparation and Implementation and Sustainment
Engage consumers	39	Intervene with patients/consumers to enhance uptake and adherence	Develop strategies with patients to encourage and problem solve around adherence	Preparation, implementation and Sustainment
	41	Involve patients/consumers and family members	Engage or include patients/consumers and families in the implementation and sustainment efforts	All phases
	50	Prepare patients/consumers to be active participants	Prepare patients/consumers to be active in their care, to ask questions, and specifically to inquire about care guidelines, the evidence behind clinical decisions, or about available evidence-supported treatments	All phases
	69	Use mass media	Use media to reach large numbers of people to spread the word about the clinical innovation	Implementation and Sustainment
	1	Access new funding	Access new or existing money to facilitate the implementation and/or sustainment	All phases
Utilize financial strategies	2	Alter incentive/allowance structures	Work to incentivize the adoption, implementation and sustainment of the clinical innovation	Preparation and Implementation and Sustainment
	3	Alter patient/consumer fees	Create fee structures where patients/consumers pay less for preferred treatments (the clinical innovation) and more for less-preferred treatments	Preparation, implementation and sustainment
	28	Develop disincentives	Provide financial or professional disincentives for failure to implement or use the clinical innovations	Preparation and Implementation and Sustainment
	34	Fund and contract for the clinical innovation	Governments and other payers of services issue requests for proposals to deliver the innovation, use contracting processes to motivate providers to deliver the clinical innovation, and develop new funding formulas that make it more likely that providers will deliver and sustain the innovation	Preparation and Implementation and Sustainment
	42	Make billing easier	Make it easier to bill for the clinical innovation	Preparation, implementation and sustainment
	49	Place innovation on fee for service lists/formularies	Work to place the clinical innovation on lists of actions for which providers can be reimbursed (*e.g*., a drug is placed on a formulary, a procedure is now reimbursable)	Preparation, implementation and sustainment
	66	Use capitated payments	Pay providers or care systems a set amount per patient/consumer for delivering clinical care	Preparation, implementation and sustainment
	70	Use other payment schemes	Introduce, review and update payment approaches (in a catch-all category) to support implementation and sustainment of the innovation	Preparation, implementation and sustainment
	9	Change accreditation or membership requirements	Strive to alter accreditation standards so that they require or encourage use of the clinical innovation. Work to alter membership organization requirements so that those who want to affiliate with the organization are encouraged or required to use the clinical innovation	Preparation, implementation and sustainment
Change infrastructure	10	Change liability laws	Participate in liability reform efforts that make clinicians more willing to deliver the clinical innovation	Preparation, implementation and sustainment
	11	Change physical structure and equipment	Evaluate periodically current configurations and adapt, as needed, the physical structure and/or equipment (e.g., changing the layout of a room, adding equipment) to best accommodate the targeted innovation	Preparation and Implementation and Sustainment
	12	Change record systems	Change records systems to allow better assessment of implementation or clinical outcomes	Preparation and Implementation and Sustainment
	13	Change service sites	Change the location of clinical service sites to increase access	Preparation and Implementation and Sustainment
	22	Create or change credentialing and/or licensure standards	Create an organization that certifies clinicians in the innovation or encourage an existing organization to do so. Change governmental professional certification or licensure requirements to include delivering the innovation. Work to alter continuing education requirements to shape professional practice toward the innovation	Preparation and Implementation and Sustainment
	44	Mandate change	Have leadership declare the priority of the innovation and their determination to have it implemented and sustained	Preparation and Implementation and Sustainment
	62	Start a dissemination organization	Identify or start a separate organization that is responsible for disseminating and supporting the ongoing delivery of the clinical innovation. It could be a for-profit or non-profit organization	Preparation and Implementation and Sustainment

NB: Categories, strategies and definitions are per the existing ERIC taxonomy (8, 13) with sustainment wording changes highlighted in red.

Sustainability research has been identified as a priority area within implementation science (8). Sustainability has been defined as “(*1) after a defined period of time, (2) the program, clinical intervention, and/or implementation strategies continue to be delivered and/or (3) individual behavior change (i.e., clinician, patient) is maintained; (4) the program and individual behavior change may evolve or adapt while (5) continuing to produce benefits for individuals/systems”* (22). A 2020 review by Moullin et al. (23) did however highlight that a number of other conceptual distinctions have been made in the field, particularly in relation to sustainment that is the “sustained use of an EBI” vs. sustainability the “sustained benefits of an EBI.” The sustainment of EBIs is critical as premature ceasing of EBIs may mean that the potential public health and clinical healthcare benefits cease or may not be achieved (24). Additionally, if EBIs are not sustained there is a significant waste of public health and clinical resources utilized for initial implementation which may have implications for reducing trust of research/academic institutions (24–26).

Whilst there is growing research focused on sustainment as an outcome (27) including consideration of specific factors (24, 27–31) associated with sustainment that may be distinct from those that matter for implementation (32, 33) the field is bereft of evidence of the most effective strategies to support the sustainment of EBIs (24, 27). A 2019 review of strategies used to sustain public health interventions identified only six studies that purposefully set out to sustain an EBI (27). Overall only nine sustainment strategies were reported with “ongoing funding,” “booster training,” “supervision and feedback” being the most frequently reported. However, there was insufficient evidence to determine the effectiveness of any one strategy in impacting sustainment. The review reported that most strategies were inadequately described providing very little detail which would enable replication. Such vague and incomplete descriptions of strategies is a limitation of the current evidence base, and highlights the need for a compilation that adequately addresses strategies that support sustainment to ensure they are consistently defined and reported. The review also emphasized the importance of sustainment being considered from the outset of a project and the need for identifying sustainment-focused strategies during the planning of an EBI. Furthermore, strategies relevant to early phases of the initial implementation process are also likely to hold relevance and lay the foundation for longer-term sustainment. However, there is currently no guidance on which strategies should be enacted, and at which phases, to best sustain an EBI.

Given that there are existing compilations for implementation strategies, it is possible that they could be extended or clarified to specifically address sustainment. However key to designing future interventions is the selection of strategies which best addresses the contextual determinants i.e., the barriers and facilitators that impede or promote (4) the sustainment of EBIs (34). While there may be some overlap with the barriers and facilitators to adoption, implementation, and sustainment of EBIs (e.g., organizational culture and resources), it is likely that there are also barriers and facilitators to sustainment of EBIs (e.g., changes in socio-political environment and funding structures) that may be distinct (35). Existing compilations may therefore be lacking in identifying and describing strategies that are specific to and necessary for sustaining an EBI. It is however acknowledged that the sustainment of an EBI is inextricably impacted by strategies selected during the previous adoption and or implementation phases (36, 37). For example, the sustainment of an EBI may be hindered if the adoption and implementation phase has relied on researchers to deliver the intervention, without consideration given to the infrastructure needed to deliver the EBI once research funding ends. Therefore, strategies for the sustainment of EBIs should be considered and planned for in unison with strategies for implementation for any progress to be made in this area. To do this compilations of implementation strategies could specifically incorporate issues relevant to sustainment. This may include updating existing implementation strategies to directly address sustainment or including new strategies that target sustainment-specific barriers and facilitators. Furthermore, whilst frameworks such as the Consolidated Framework for Implementation Research (CFIR) (38) are useful to identify what factors may influence sustainment they do not address how or when change needs to occur (39). Therefore if we are to plan for sustainment at the beginning of implementation efforts, as has been recommended (36), direction on which strategies need to be employed during which phase of the implementation process is needed.

This research is still in its infancy, and there is an opportunity to establish the use of a compilation of sustainment strategies to allow for consistent reporting and, ultimately, empirical testing. As it is likely that sustainment strategies need to be considered during all phases of implementation, extending an existing compilation of implementation strategies that is already widely used, is likely to support the consideration of sustainment at appropriate phases of implementation and avoid unnecessary duplication. Thus, the aim of this study is to adapt, refine and extend an existing compilation of implementation strategies (ERIC) (13, 21) to explicitly incorporate sustainment, as well as specify the phases of implementation that such strategies are likely to be most salient according to the Exploration, Preparation, Implementation and Sustainment (EPIS) (40) framework.

## Materials and methods

### Adapting and extending the ERIC compilation to incorporate sustainment

A two-phase iterative approach to adapt the ERIC compilation to include sustainment was undertaken, based on procedures similar to those previously used in the development (41) or adaptation (42) of ERIC or other taxonomies. This involved:

#### Adapting and extending through consensus

Consistent with other approaches to developing and extending the ERIC compilation (13, 21, 42), we convened a team of 11 researchers, policy-makers, and practitioners (co-authors of this paper) from Australia, Canada and The United States, who undertook an iterative process of reviewing and adapting the current compilation to incorporate strategies specific to sustainment. For the purpose of this study we defined sustainment as “*the sustained use or delivery of an intervention in practice following cessation of external implementation support”* (26, 36). The team are experts in implementation and or sustainability science, and or health service delivery, and included two of the original authors of the ERIC compilation (BP and TW) an expert on the conceptual distinction of ERIC strategies (13, 21, 34). Both BP and TW have adapted the ERIC for specific contexts (42, 43). In order to adapt and extend the ERIC the following steps were undertaken.

##### Step 1: Barriers to sustainment

We first identified barriers to sustainment from existing studies. These nine publications (27–29, 44–49) were found through snowballing for literature of “barriers to sustainment” which a research assistant extracted into an excel spreadsheet.

##### Step 2: Mapping ERIC strategies to address key barriers

To help identify where wording changes may be needed or where additional strategies may need to be created two authors (AH and NN) independently mapped these barriers to existing ERIC strategies. Where the authors felt that a barrier could not be adequately linked to an existing ERIC strategy, they independently drafted proposed wording changes to an existing strategy or identified if a new strategy was needed. The two authors then met to discuss coding, suggested wording changes and or new strategies until they reached consensus. A third author (BP) then reviewed, provided feedback and then met with AH and NN to discuss revisions until consensus was reached.

##### Step 3: Iterative consensus process

Following completion of Step 2 all team members were asked to independently review the suggested wording changes and the proposed new strategies developed by AH, NN and BP. They were specifically asked to review and document any edits they believe should be made, or any disagreements they had with the current suggestions, along with detail of their reasoning. After each iteration AH and NN reviewed all feedback. Where there were instances of disagreement between authors they met to develop a proposed amendment and circulated this to all authors for their review. This process of review and updating by the entire team continued for three rounds until consensus was reached.

#### Preliminary application of the sustainment-explicit glossary

Following the above, the authors undertook a preliminary test of the application and logic of the sustainment-explicit ERIC glossary to determine its ease of application in the field of sustainment, and if any further adaptions or amendments were needed. As this is still an emerging field to identify potential trials which have employed sustainment strategies we reviewed the National Institutes of Health (NIH) database of trials funded in 2019. We also searched the table of contents of the leading implementation science journals, which included: *Implementation Science, Implementation Science Communications*, and *Frontiers in Public Health* for sustainment interventions published between 2018 and 2020. Overall, 12 trials or protocols were identified. As our goal was to check the logic of our proposed adaptation we randomly selected a small number of these studies (*n* = 6) to test the sustainment-explicit glossary. Two authors (AH and NN) independently coded the strategies described in those publications against those in the sustainment-explicit ERIC glossary. The authors then compared coding to identify areas of confusion, disagreement, or if any additional strategies emerged. This process was designed to identify where updates were needed to improve the content or wording of the glossary and ensure feasibility in its application. The final glossary was reviewed and agreed on by all authors involved.

### Implementation phase and strategy utility

To help researchers and practitioners identify when they might consider employing each strategy, we categorized each strategy against the phase(s) of implementation according to the Exploration, Preparation, Implementation and Sustainment (EPIS) Framework (37). To complete this categorization, the same iterative process described above was followed. EPIS was selected as a guiding taxonomy, as it is a widely used and provides clear definitions for each phase. Definitions of the EPIS as defined by the developers (40) were provided to co-authors to help them code the ERIC strategy to the EPIS phase(s).

## Results

The sustainment explicit ERIC glossary is presented in Table 1.

### Adapting ERIC definitions

Of the 73 ERIC strategies, the definitions of 45 were amended to make sustainment more explicit. For the majority (*n* = 41) this involved minor surface level changes to include the words “sustainment” or “sustainability.” For example, the definition of “Centralized Technical Assistance” was changed to “*develop and use a centralized system to deliver technical assistance focused on implementation and sustainment issues.”* Other surface level changes to definitions were more elaborative. For example, the definition of “Promote Adaptability” was changed to “*Identify the ways a clinical innovation can be tailored to meet local needs and clarify which elements of the innovation must be maintained to preserve fidelity. Continue to assess and adapt the fit of the innovation to ensure that it is appropriate and sustained if still relevant.”*

The other four strategies where adaptations were made were identified as being in need of slightly deeper level adaptations. These deeper level adaptations were extensions of existing strategies and reflect changes made to the substance of the definition (42), to specifically encompass issues of sustainment, typically because the original definition more explicitly focused on the application of the strategy at an earlier phase of implementation. For example *Obtain formal commitments* (strategy 47) was defined as “*Obtain written commitments from key partners that state what they will do to implement the innovation and how they will support sustainment if it has the intended beneficial effects”* however it was acknowledged that this didn't accurately capture a key barrier to sustainment in regards to ongoing support or decisions around continuation. Accordingly *Re-affirm formal commitments* (an extension of strategy 47) was added which was defined as “*Revisit the written commitments obtained from key partners that state what they will do to implement and sustain the innovation. Assess whether these commitments are being upheld and whether new commitments are required to help sustain the innovation.”* The additional strategies are: *Review and update educational materials* (extension of strategy 29); *Identify successful sustainers* (extension of strategy 36); *Re-engage with local opinion leaders* (extension of strategy 38); *Re-affirm formal commitments* (extension of strategy 47). See Table 1 for the detailed definitions of these strategies.

### Novel sustainment strategies

One new sustainment focused strategy was identified: *Communicate with stakeholders the continued impact of the EBP*. This strategy takes the information obtained from *Audit and provide feedback* and/or *Develop and organize quality monitoring systems* strategies and communicates data to external stakeholders, end-users, and consumers to demonstrate the ongoing benefit, cost effectiveness, or return on investment of the innovation with continued implementation. Conceptually, this strategy seems to fit within the ERIC *Use evaluative and iterative strategies* cluster (21).

### Preliminary application of the sustainment-explicit ERIC glossary

Application of the sustainment-explicit ERIC identified wide variation in detail and language used to describe the specific strategies employed in the reviewed studies. Consequently, following the initial independent review by the two authors, a thorough discussion and joint application was undertaken to help identify any gaps or areas in need of improvement in the compilation. No new strategies were identified through the coding of published sustainment trials or manuscripts that needed to be considered for inclusion in the glossary. Minor wording changes were made to help clarify some of the strategies and how they relate to sustainment to ensure consistency in interpretation and application.

### Implementation phase and strategy utility

Table 1 shows that the majority of strategies (*n* = 44) were identified as being relevant for consideration during three of the four phases of the EPIS Framework, with 43 of the 44 likely to be needed during preparation, implementation and sustainment phases. Only five strategies were identified as being *only* relevant during the sustainment phase, which were the four that received deeper levels of adaptation to focus on sustainment (noted above) as well as the novel strategy (also noted above). Thus, majority of existing ERIC strategies were viewed as relevant for more than one EPIS phase, including sustainment.

## Discussion

This is one the first of studies to systematically evaluate an existing compilation of implementation strategies for their relevance for supporting the sustainment of evidence-based programs. The two-phase iterative approach resulted in superficial wording changes to the definitions of 41 of the 73 existing ERIC strategies, slightly deeper wording changes to four ERIC strategies, and the addition of one new strategy. The study also provides guidance to researchers and implementation support practitioners looking to design implementation or sustainment interventions by identifying the phase, according to EPIS framework, when the strategy may need to be considered and employed. It is hoped that a sustainment-explicit glossary based on an existing compilation of implementation strategies will encourage and support those undertaking implementation research to explicitly consider sustainment from the outset and to use a common language when planning and describing their research and practice.

Whilst others have adapted or applied the ERIC compilation to be relevant to a particular setting (42) or class of interventions (50), or to advance understanding of a particular subset of strategies (51), our sustainment-explicit ERIC glossary required minimal changes. We were able to include sustainment concepts by making no changes to strategy names, minimal modifications to definitions and identified only one new strategy. Our extensive mapping exercise of the ERIC strategies to known barriers and facilitators of sustainment from a broad range of studies in clinical and community settings (27–29, 44–49) and sustainability frameworks (24, 36, 52), ensured that we were adequately capturing strategies specific to addressing the main barriers to sustainment.

The preliminary application of the glossary further highlighted the lack of standardized reporting that is already emerging within the sustainment literature. Of the studies reviewed (*n* = 6), many of the strategies utilized were not adequately described in enough detail, or were hard to disentangle from other strategies, which would make it difficult for any future studies wishing to synthesize the effects of these strategies. To avoid the challenges that this has caused historically in the field of implementation science, we implore those planning, or currently undertaking, sustainment research to use consistent terminology to describe their chosen strategies, particularly when multiple strategies are used. Furthermore, as recommended by Michie and Johnston (53) for implementation interventions, we encourage trialists to describe these strategies with sufficient detail in terms of “what,” “who,” “when,” “where” and “how,” so these components of each strategy can be sufficiently understood and replicated by others. Frameworks such as those developed by Proctor et al. (8) or Presseau et al. (54) provide useful guidance for specifying this behavior (in the context of implementation and sustainment interventions) (1). If strategies addressing sustainment are consistently described in future research trials this will enable replication studies to be undertaken and study findings synthesized to identify effective strategies or combinations of strategies, and the optimal timing of their delivery, all of which will enhance the design of future sustainment interventions. Whilst the sustainment-explicit ERIC glossary captures all strategies previously identified (27), as evidence in the field continues to grow there may be a need for new strategies to be added. Therefore, this glossary will need to be continuously refined to maintain its utility in sustainment research.

Our application of the EPIS Framework found that a large majority of strategies should be considered during the design and earlier phases of implementation. This is consistent with others who have advocated that implementation and sustainment are interconnected and therefore need to be planned for in advance (55–58). This is also supported by more recent sustainability frameworks such as the Dynamic Sustainability Framework or the RE-AIM extension for sustainment which posits sustainability is not “static,” but rather dynamic, impacted by the changing context in which the intervention is being delivered, the evolving scientific evidence, and the dynamic needs of a population. In a recent study the original developers of the ERIC assessed which strategies experts perceived as being most essential for implementation of three high priority mental health care practices in the US Department of Veteran Affairs (43). The authors found that experts consistently selected a similar set of ERIC strategies as essential for implementation success, regardless of type of EBI (43) or implementation phase. Again, this study highlights the interconnectedness of sustainment with the earlier phases of implementation, and how strategies can be perceived as relevant across the different implementation phases. Shelton et al. (36) suggests that in planning for sustainability, monitoring the reach, adoption, effectiveness and implementation of an EBI is essential to identify early on when challenges are arising and if and how strategies can be adapted, refined, or introduced to support the sustainment of the EBI and address health inequities that may be exacerbated over time.

Robust and valid frameworks or theories specific to sustainability such as the Dynamic Sustainability Framework (52) or the Integrated Sustainability Framework (24) should be employed alongside the sustainment-explicit ERIC glossary, when planning sustainment trials. These frameworks and theories will help identify issues specific to sustainment that should be addressed by any strategies being developed and evaluated (59). Unfortunately, a large proportion of sustainability research is not based on relevant theories, frameworks, or models and for those studies that have, there is wide variation and limited validity in the theories and frameworks commonly applied (59). There is significant need for sustainability research to evaluate the application of sustainability frameworks alongside a compilation such as ERIC (60). This is important if we are to identify how or why strategies impacting sustainment exert their effects (i.e., the mechanisms through which they work) (6). Once this is known we may improve the effectiveness and cost-effectiveness of future interventions by keeping, strengthening, adding or removing strategies that target (or don't) mediators which lead to improvements in sustained implementation (5, 61).

There are several limitations to this study. First, unlike the methods used to develop the original ERIC compilation, we only had a small number of implementation and sustainability experts (*n* = 11) convened to specifically work on this project. Whilst we represented community and clinical perspectives from various countries to gain a broader perspective on this issue, a larger, more diverse, group of experts should further review and revise this glossary for use in sustainment-focused work. Second, we only tested the application of the glossary with a small number of studies. This was undertaken as to test the logic of the amendments; it was not designed to be an extensive application of the sustainment-explicit ERIC or to identify what strategies are being used in sustainment trials. Accordingly, this glossary has not been extensively tested, further application and review of this glossary is needed and welcomed and through its use, it may be evident that further updates are required. Finally, ongoing work is needed to assess the extent to which the sustainment-explicit ERIC glossary is relevant to low- and middle-income countries (62), as this study did not explicitly address this question.

## Conclusions

The sustainment-explicit ERIC glossary addresses the need for explicit and clear definitions of strategies to be used in sustainment interventions. The application of relevant strategies during planning and implementation phases may subsequently enhance the evidence-base for the field, and ultimately the sustainment, spread and scale of interventions and improvements in our communities health (63). Future work is needed to empirically test the effectiveness of these strategies in sustaining EBIs in clinical and community settings.

## Data availability statement

The original contributions presented in the study are included in the article. Further inquiries can be directed to the corresponding author.

## Author contributions

NN obtained funding for the study. NN, AH, BP, and LW conceived the study concept and developed the study design. NN and AH undertook initial adaptations of the ERIC and classification against EPIS. BP and TW provided expert advice as original developers of ERIC. RCS, CL, LW, MH, SY, RS, and MK advised on and undertook the adaption, extension, consensus process, and pilot testing of the tool. NN and AH developed the draft manuscript. All authors contributed to the article and approved the final version of the manuscript.

## Funding

This project is funded through the NHMRC as part of NN's Medical Research Future Fund (MRFF) Investigator Grant (GS2000053), BP is supported in part through the U.S. National Institutes of Health (K01MH113806, R01CA262325, P50CA19006, and P50MH126219) and the Agency for Healthcare Research and Quality (R13HS025632), RS is supported by an NHMRC Medical Research Future Fund (MRFF) Investigator Grant (G2000052), LW is supported by an NHMRC Investigator Grant (G1901360), and SY is supported by an ARC DECRA (G1600359). The funders had no role in the study design, conduct of the study, analysis, or dissemination of findings.

## Conflict of interest

The authors declare that the research was conducted in the absence of any commercial or financial relationships that could be construed as a potential conflict of interest.

## Publisher's note

All claims expressed in this article are solely those of the authors and do not necessarily represent those of their affiliated organizations, or those of the publisher, the editors and the reviewers. Any product that may be evaluated in this article, or claim that may be made by its manufacturer, is not guaranteed or endorsed by the publisher.

## Abbreviations

EBI: Evidence Based Intervention
EBP: Evidence Based Practice
EPIS: Exploration, Preparation, Implementation and Sustainment
ERIC: Expert Recommendations for Implementing Change.
